# Conventional Scalpel Technique for Frenectomy During Orthodontic Treatment: A Case Report

**DOI:** 10.7759/cureus.60252

**Published:** 2024-05-14

**Authors:** Sanehi D Punse, Prasad V Dhadse, Shrishti S Salian, Ruchita T Patil

**Affiliations:** 1 Department of Periodontics and Implantology, Datta Meghe Institute of Higher Education and Research, Wardha, IND

**Keywords:** periodontal plastic surgery, conventional scalpel frenectomy, midline diastema, frenectomy, frena

## Abstract

Frenum aberrations in the maxillary and mandibular regions are pivotal concerns, particularly regarding midline diastema and gingival health. The frenum is composed predominantly of collagenous and elastic fibers. There are various frenal attachment anomalies that may result in gingival recession. Amidst treatment options, conventional scalpel frenectomy emerges as a viable solution, showcasing its efficacy in addressing deviant frena. Ultimately, our findings underscore the imperative for personalized interventions to alleviate aesthetic apprehensions and uphold periodontal integrity in adult populations.

## Introduction

The pursuit of a beautiful smile has made acquiring dental care crucial due to aesthetic concerns [1]. The persistent midline diastema in maxillary incisors has been deemed an aesthetic issue. One of the etiological causes of midline diastema is the appearance of an abnormal frenum [2]. The ectolabial bands that join the palatine papilla to the upper lip tubercle as the tubercle erupts give rise to the maxillary labial frenum. When the mandibular frenum is accompanied by a reduced vestibular level and insufficient width of the attached gingiva, it is regarded as abnormal [1]. During various phases of growth and development, there might be changes in the size of the frenum, form, and location. Its size tends to decrease as it grows. Typically, broad and thick in early childhood and turn smaller and thinner as they get older [1].

A clinical and morphological grouping for the maxillary frenal attachment was created based on the anatomical attachment site given by Placek et al. in 1974. According to the location of frenum attachment, they divided the attachment into four distinct groups: mucosal, gingival, papillary, and papilla penetrating [3]. This is found to be related to loss of papilla, diastema, recession, misaligned teeth, difficulty in brushing, and improper denture fit. When the frena (derived from the Latin word frenum) adheres firmly to the gingival margin which can be due to an obstruction to the correct toothbrush placement or by the opening of the gingival crevice due to a muscular pull, the gingival health may also be compromised by a gingival recession [4].

By placing stress across the frenum and observing the shifting of the papillary tip or blanching those results from localized ischemia, one can clearly locate the frenum. When it is abnormally wide and there is no visible area of attached gingiva along the midline or when the interdental papilla moves when the frenum is expanded, it is classified as pathogenic [5]. Techniques called frenectomy or frenotomy can be employed to rectify the aberrant frenum. Although a frenotomy involves making an incision to relocate the frenal attachment, a frenectomy entails eliminating the frenum entirely, involving its connection to the bone underneath. A traditional scalpel frenectomy was performed in this case. The surgical incision is sutured and sealed after the entire band of tissue and its alveolar connection are removed.

## Case presentation

**Figure 1:**
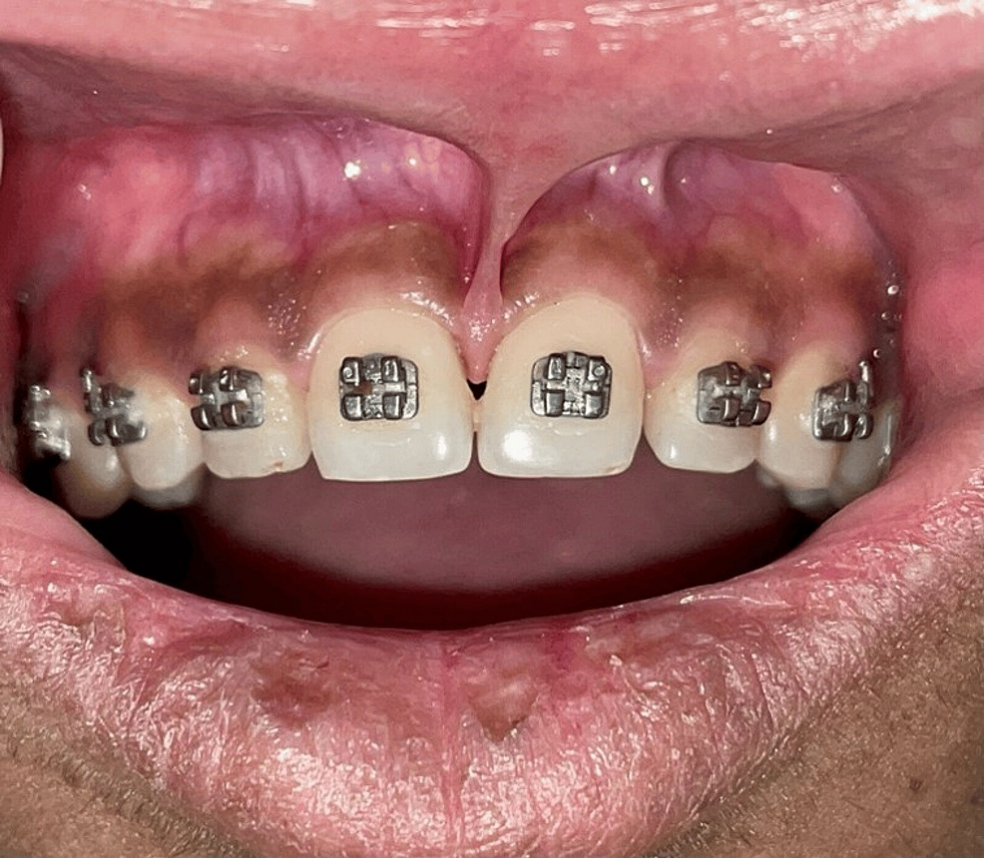
Pre-operative photograph showing high maxillary frenum attachment.

A male patient of age 21 was referred by the Department of Orthodontics to the Department of Periodontics. The patient was receiving orthodontic treatment for a pre-orthodontic midline diastema, which was closed with high papillary-type frenal attachment between two maxillary central incisors (Figure 1).

A comprehensive medical history was obtained in order to exclude minor conditions. The necessary surgical operation was first explained to the patient, and then a signed agreement was obtained. Following a standard hematological assessment, the results fell in the expected range (Table 1).

**Table 1:** Hematological reports Hb: Hemoglobin; BT: bleeding time; CT: clotting time

Hematology	Finding value	Normal value
Hb%	12.5 g%	M- 12-15.5 g%; F- 11-14.5 g%
BT	2 min 00 sec	1-3 min
CT	2 min 30 sec	1-5 min

**Figure 2:**
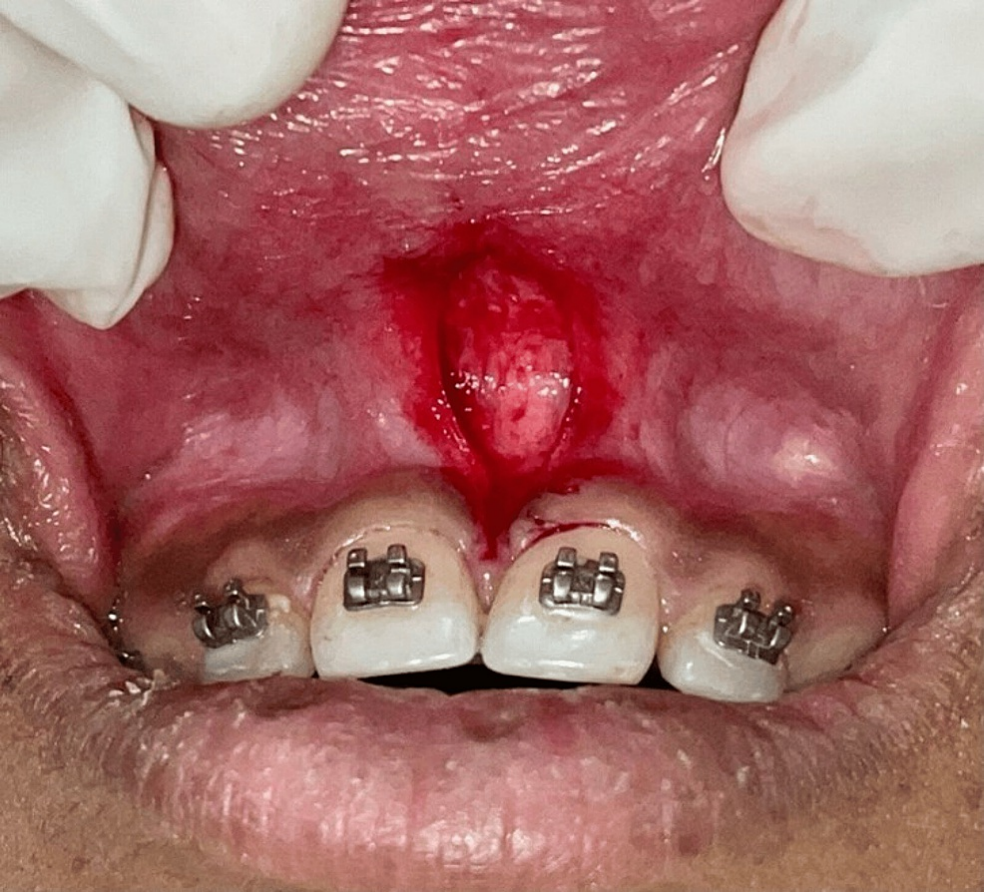
A V-shaped incision was given and the entire band of tissue with its alveolar attachment was removed.

2% lignocaine combined with 1:80,000 adrenaline (LOX 2% Adrenaline) was used to anesthetize the affected zone. Using a #15 blade, the entire band of tissue was held using hemostat, along with its alveolar attachment was removed from the frenum (Figure 2).

**Figure 3:**
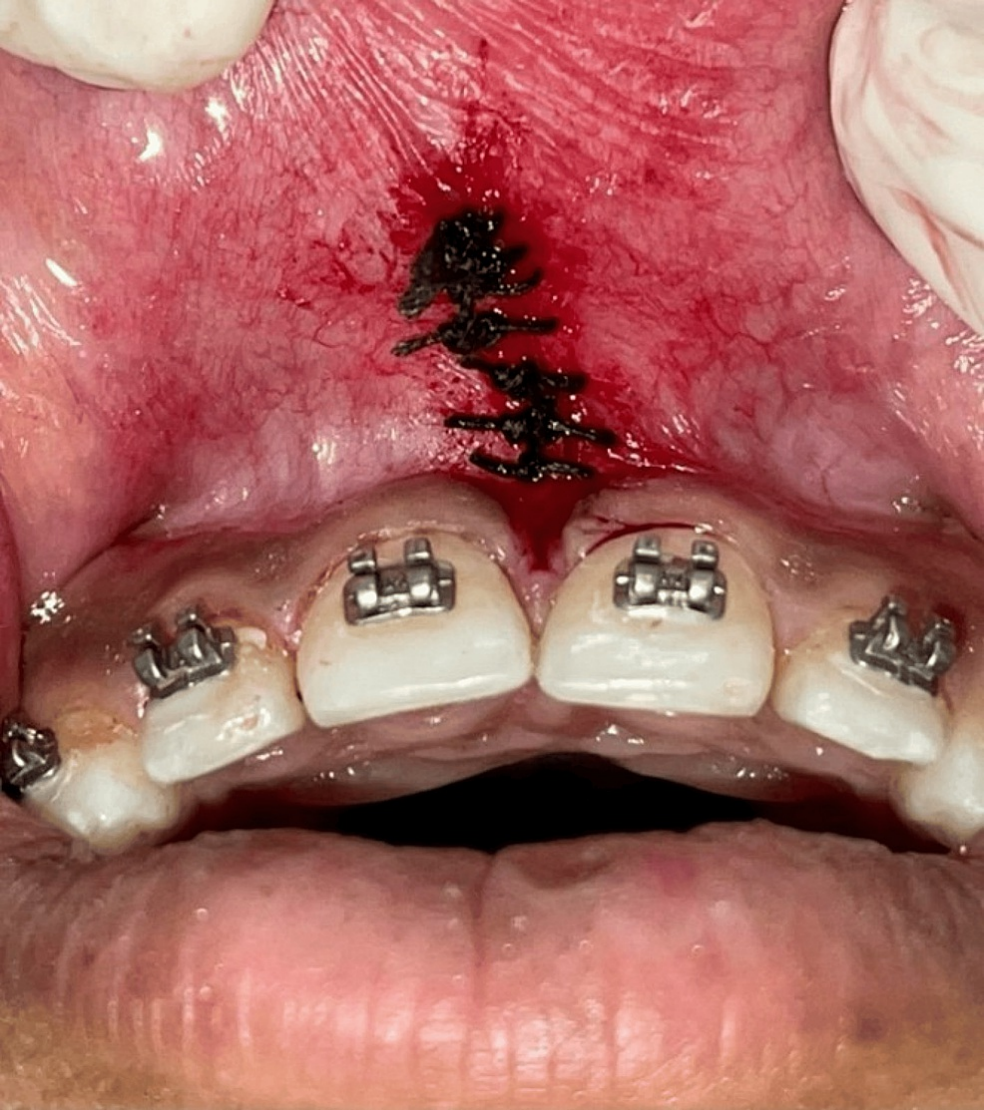
Simple interrupted sutures were placed.

Following the release of any residual fibrous adhesions to the periosteum beneath the wound, 3-0 silk-interrupted sutures were used to seal it (Figure 3). The region was coated with COE-PAK, a periodontal dressing.

**Figure 4:**
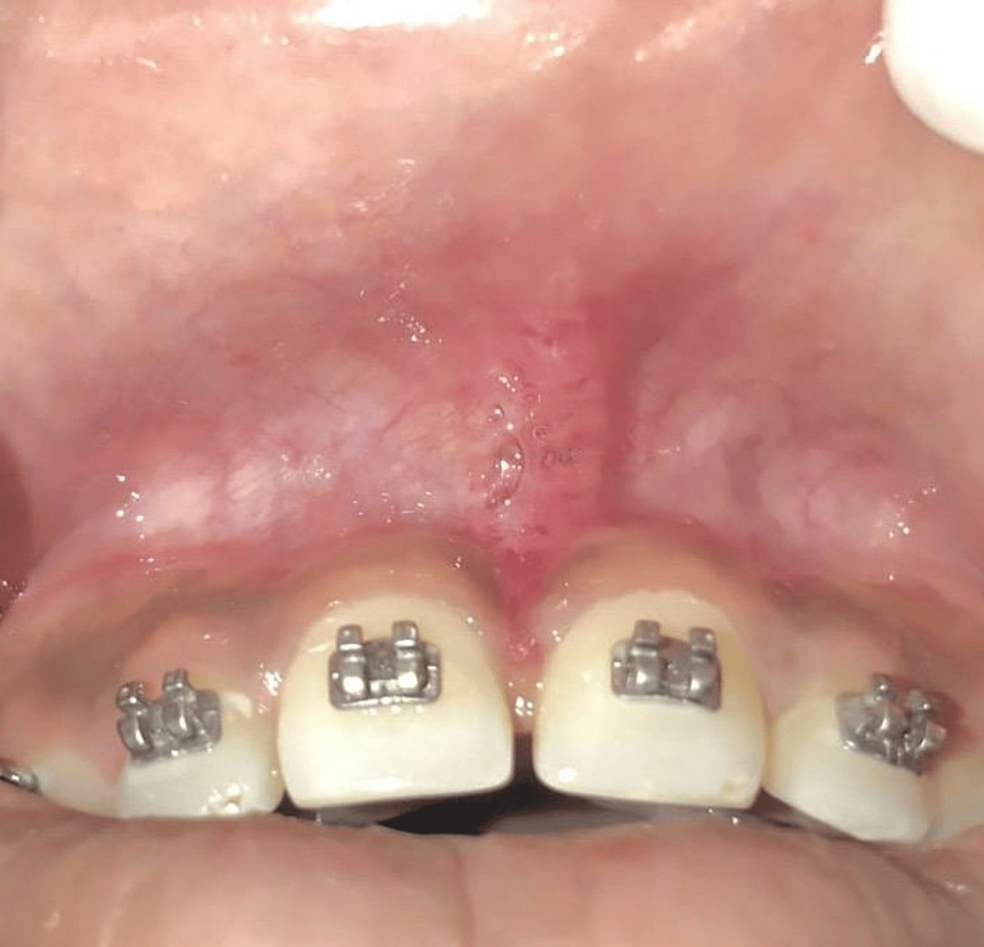
Post-operative view with satisfactory healing was seen after one-month follow-up.

On the seventh day, the patient was brought back for suture removal. At a one-month follow-up, a labial frenectomy using a traditional scalpel technique showed complete and excellent healing without scarring and favorable outcomes maintained with regard to ortho-perio assessment (Figure 4).

## Discussion

However, the generally used method for frenectomy that prevails is the classical technique, despite the numerous changes that have been suggested. In 1961 and 1964, Archer and Kruger introduced the classical technique [2]. In instances of midline diastema with an abnormal frenum, this procedure is used to guarantee the removal of the muscle fibers thought to be linking the palatine papilla and orbicularis oris.

Multiple alterations have been implemented to address the problems resulting from an abnormal labial frenum. In order to eliminate the traditional diamond-shaped scar and speedy recovery, other surgical procedures have recently been developed, including frenal displacement by Z-plasty, frenectomy with soft-tissue transplant, and laser treatments. Every technique has benefits and drawbacks of itself [6,7]. However, the majority of procedures rely on surgical frenectomy procedures, such as the conventional (classical) technique, and do not consider appearances or the area of the attached gingiva. Miller's unilateral pedicle flap technique and the outcomes of a frenectomy with a bilateral pedicle flap are shown. As primary intention healing occurs, the pedicle flap frenectomy procedure provides more aesthetic outcomes, uniform color, improved gingival attachment, and no creation of visible scars [8,9]. However, there are a lot of benefits to using a scalpel. It is unique in that it is accurate, affordable, and easy to use. Surgeons value its remarkable control and capacity to maintain its structural integrity when performing surgeries. Additionally, it is linked to better wound-healing results.

However, the drawbacks of using a scalpel are higher unesthetic requirements, the need for suturing, inadequate hemostasis, and unfavorable complications following surgery like pain, edema, and unease [10]. Nevertheless, these problems can be avoided with an accurate and careful approach. A variant of the different methods is conventional frenectomy, which seeks to reposition the frenum and improve outcomes.

## Conclusions

In the presented case, a typical scalpel frenectomy procedure, a cornerstone of periodontal plastic surgery, was used to efficiently remove a high labial frenum attachment. The postoperative outcome demonstrated remarkable healing with no evidence of hypertrophic development of scars, fulfilling both the doctors' and the patients' demands for practical and aesthetic benefits. Regardless of the technical challenges, the scalpel frenectomy procedure produced favorable outcomes, especially in the successful removal of the papilla-piercing frenum, which aided greatly in the achievement of orthodontic treatment.

The positive results, in this case, demonstrate the usefulness and dependability of the traditional scalpel frenectomy approach in treating high labial frenal attachments. Its capability to provide optimal functional and aesthetic outcomes, combined with a low risk of postoperative problems such as hypertrophic scarring, solidifies its place as an ideal option for frenectomy treatments. In summary, the case's favorable outcome suggests that the scalpel frenectomy treatment will continue to be useful and relevant in the real world for treating orthodontic patients' various needs.
